# Floral traits influence pollen vectors’ choices in higher elevation communities in the Himalaya-Hengduan Mountains

**DOI:** 10.1186/s12898-016-0080-1

**Published:** 2016-05-24

**Authors:** Yan-Hui Zhao, Zong-Xin Ren, Amparo Lázaro, Hong Wang, Peter Bernhardt, Hai-Dong Li, De-Zhu Li

**Affiliations:** Key Laboratory for Plant Diversity and Biogeography of East Asia, Kunming Institute of Botany, Chinese Academy of Sciences, Kunming, 650201 People’s Republic of China; Kunming College of Life Sciences, University of Chinese Academy of Sciences, Kunming, 650201 People’s Republic of China; Mediterranean Institute for Advanced Studies, c/Miquel Marquès 21, 07190 Esporles, Spain; Department of Biology, Saint Louis University, Saint Louis, 63103 MO USA; Germplasm Bank of Wild Species, Kunming Institute of Botany, Chinese Academy of Sciences, Kunming, 650201 People’s Republic of China

**Keywords:** Diversity of pollen vectors, Floral display, Flower abundance, Flower shape, Flower size, Flowering duration, Plant-pollen vector interactions, Pollen vector density, Plant selectiveness, Specialization

## Abstract

**Background:**

How floral traits and community composition influence plant specialization is poorly understood and the existing evidence is restricted to regions where plant diversity is low. Here, we assessed whether plant specialization varied among four species-rich subalpine/alpine communities on the Yulong Mountain, SW China (elevation from 2725 to 3910 m). We analyzed two factors (floral traits and pollen vector community composition: richness and density) to determine the degree of plant specialization across 101 plant species in all four communities. Floral visitors were collected and pollen load analyses were conducted to identify and define pollen vectors. Plant specialization of each species was described by using both pollen vector diversity (Shannon’s diversity index) and plant selectiveness (d’ index), which reflected how selective a given species was relative to available pollen vectors.

**Results:**

Pollen vector diversity tended to be higher in communities at lower elevations, while plant selectiveness was significantly lower in a community with the highest proportion of unspecialized flowers (open flowers and clusters of flowers in open inflorescences). In particular, we found that plant species with large and unspecialized flowers attracted a greater diversity of pollen vectors and showed higher selectiveness in their use of pollen vectors. Plant species with large floral displays and high flower abundance were more selective in their exploitation of pollen vectors. Moreover, there was a negative relationship between plant selectiveness and pollen vector density.

**Conclusions:**

These findings suggest that flower shape and flower size can increase pollen vector diversity but they also increased plant selectiveness. This indicated that those floral traits that were more attractive to insects increased the diversity of pollen vectors to plants while decreasing overlap among co-blooming plant species for the same pollen vectors. Furthermore, floral traits had a more important impact on the diversity of pollen vectors than the composition of anthophilous insect communities. Plant selectiveness of pollen vectors was strongly influenced by both floral traits and insect community composition. These findings provide a basis for a better understanding of how floral traits and community context shape interactions between flowers and their pollen vectors in species-rich communities.

**Electronic supplementary material:**

The online version of this article (doi:10.1186/s12898-016-0080-1) contains supplementary material, which is available to authorized users.

## Background

Studies show that specialization of plant species within a community ranges from extremely generalized to highly specialized [1, 2]. Community level investigations on how floral traits influence plant specialization provide us with a broader understanding of floral trait evolution and adaptation [3–10]. Plant species with larger flowers/inflorescences tend to attract a greater diversity of pollinators [3, 11]. Larger flowers may be preferred by pollinators because they are easier to see and minimize the time foragers need to locate them [12]. In addition, flower size often correlates positively with reward amounts and production, including pollen and/or nectar [13, 14].

Differences in reward accessibility of flowers may also play an important role in the specialization of some pollination systems or syndromes. Open flowers (dish shaped with shallow floral tubes) and compacted inflorescences (e.g. head or spicate) are easily accessible to most pollinators and may attract a greater diversity of floral foragers [11, 15]. In contrast, individual flowers with bilateral symmetry (e.g. gullet or flag-shaped) [16, 17], flowers that are inverted on their pedicels [18] and flowers that produce elongated tubes or spurs with narrow sinuses [19], are more likely to restrict access to the majority of resident pollinators. These flowers are more likely to be pollinated by animals with specialized mouthparts and/or specialized modes of foraging [4, 20]. Additional studies have shown that plant species with large floral displays and remain in bloom for long periods can increase visitation frequencies and/or the number of visiting pollinator species [3, 21–23].

Floral visitation to a plant species is also related to the community composition of both plants and flower visitors [11, 24, 25]. Plant species with abundant flowers commonly interact more frequently with more pollinator species than plant species showing depauperate flowering [4, 10]. Floral visitation also increases with increasing pollinator abundance [11]. These findings were consistent with the neutrality hypothesis that states that, the occurrence of interactions results from random encounters among individual plants and pollinators [26]. Besides, the abundance and composition of co-flowering species could also influence the patterns and rates of floral visitation of a plant species [25]. Sympatric insect-pollinated plants can either compete for pollinators or mutually attract and share the same pollinators (pollination facilitation), depending on the relative abundance, accessibility and diversity of their floral rewards [24, 25, 27, 28]. In addition, the potential for an indirect influence via shared pollinators was also related to the phylogenetic distance among co-flowering species [25]. Plant and pollinator community composition often show systematic variation along elevational gradients [29, 30] and this may result in corresponding variation of pollen vector choices to co-flowering plant species.

Plant species could benefit by attracting a greater diversity of pollinators to increase reproductive success [31]. However, pollinator sharing among plant species may lead to declines in fitness due to competition for pollinators and the increased incidence of interspecific pollen transfer [32]. Although the evolution of divergence in pollen placement on pollinators’ bodies has helped to minimize interspecific pollen transfer, this mechanism does not reduce all reproductive interference [33]. Koski et al. [10] found that plants decreased overlap in their use of flower visitors by increasing flower sizes across a meta-community of five serpentine seeps in California. Therefore, to achieve optimal reproductive output, a successful strategy for pollinator-dependent plant species could be to produce enough rewards and occur at such a relatively high abundance to attract a high diversity of pollinators while decreasing the need to share pollinators.

Empirical evidence showing how floral traits and community context influence plant-pollinator interactions at the community level remains uncommon. Such studies tend to be restricted to regions where plant diversity is low [3, 5, 9, 11]. We studied the interactive effects of floral traits and pollen vector community composition in highly diverse and temperate communities within a Himalayan floristic province. Specifically, we measured floral traits and plant specialization in 101 herbaceous species found in four communities (elevation from 2725 to 3910 m) in the Yulong Mountain in Lijiang, SW China. We used two species-specific indices, pollen vector diversity (Shannon’s diversity index) and selectiveness (d’ index), to describe the specialization of each plant species. We addressed the following questions: (1) Does plant specialization differ among the four study communities? (2) How do floral traits and pollen vector community composition (i.e., pollen vectors diversity and density) influence specialization in plant species?

## Methods

### Study systems

The study was conducted on the Yulong Mountain in the Himalaya-Hengduan Mountains, SW China. The study communities were located at the Lijiang Forest Ecosystem Research Station operated by the Kunming Institute of Botany, Chinese Academy of Sciences. We selected four 1.5 ha subalpine/alpine meadows on the eastern slope of the mountain. All meadows were at high elevation but with a difference of 1185 m between the lowest and highest community: 1) Yushuizhai (YSZ), 2725 m above sea level (a.s.l.), 27°00′10″N, 100°12′05″E; (2) Haligu (HLG), 3235 m a.s.l., 27°00′09″N, 100°10′57″E; (3) Yakou (YK), 3670 m a.s.l., 27°00′56″N, 100°10′17″E; and (4) Diyifeng (DYF, above tree line), 3910 m a.s.l., 27°01′41″N, 100°11′03″E. The linear distance between neighboring communities was ca. 2.0 km. Additionally, there was variation in landscape characters based on local land use and construction. The YSZ site was adjacent to a tourist center while HLG was adjacent to a major water reservoir below the field station. Sites YK and DYF were at higher elevations with less anthropogenic impact. All sites received some grazing by cattle, yaks or horses. The vegetation cover within a 2 km radius also differed among sites from forests dominated by *Pinus* species to *Abies* and *Rhododendron* species. Flowering duration and pollinator activity periods tended to decrease with increasing elevation. Mean temperatures (from 11 May to 29 September, 2012) among the four communities varied from low to high elevation; they were 16.6, 12.9, 9.6 and 8.9 °C respectively (recorded with Temperature/Relative Humidity Data Loggers, HOBO U23-001, Onset Computer Corporation, Bourne, MA, USA).

### Field surveys of flower visitors and measurements of floral traits

We collected flower visitors from 101 insect-pollinated, herbaceous species at the four communities from early May to early October in 2012. These collections nearly covered the entire flowering periods of all four communities. Two creeping shrubs (*Cotoneaster adpressus* and *Rhododendron fastigiatum*) were found infrequently in our quadrats but we excluded them from data analyses, because both species grew as overlapping clumps and it was not possible to segregate and evaluate individual floral displays (see below). We conducted nine surveys at 2-week intervals for each community. Floral visitors were collected by walking along arranged transects approximately 150 m in length and 2 m in width from 9:00–17:00 h on either sunny days or during sunshine gaps on cloudy or foggy days. Only insects that contacted plant reproductive organs or were foraging for nectar and/or pollen were classified as legitimate visitors and collected. We calculated flower visitor density by using the average number of insect visits recorded per observation period for each survey. Insect specimens were netted and euthanized in small jars with fumes of ethyl acetate prior to pinning and identification. Voucher specimens were deposited in the Kunming Institute of Botany, Chinese Academy of Sciences.

Flower abundance, floral display and flowering duration were estimated by using 30, 1 × 1 m^2^ quadrats at each community. These quadrats were spaced within the 1.5 ha plot. The minimum distance between two neighboring quadrats was 10 m. The number of flowering individuals per species and the number of open flowering units produced by each individual (floral display, hereafter) inside the quadrats were recorded in each survey. Flowering units were defined as either individual flowers or whole inflorescences depending on species. For species with densely compact inflorescences (e.g. Asteraceae and Apiaceae) each inflorescence was counted as a single flowering unit [22]. We used the mean number of flowering units of each survey to describe flower abundance of a plant species. Floral display of each species was defined as the mean number of flowering units per individual in each survey. The flowering duration of a species was defined as the number of weeks that the plant was recorded in bloom in the quadrats.

Flower and inflorescence shape for each species (referred to here as flower shape, hereafter) was subdivided into unspecialized and specialized flowers based on corolla traits or inflorescence architecture [11]. The unspecialized flowers or inflorescences were held erect, had easily accessible floral rewards in bowl-shaped perianths or in short tubes produced by single flowers (e.g. radially symmetrical members of the Rosaceae) or inflorescences (e.g. Asteraceae). Specialized flowers produced corollas that hid their rewards in elongated tubes or gullets (e.g. Lamiaceae) or spurs, restricting foragers with short mouthparts.

We used different formulae to calculate the mean flower size (unit area) of 10–20 randomly selected flowering units for each species according to the shape of flowers/inflorescences. In radially symmetrical (actinomorphic), shallow or flat flowers, and the head inflorescences of the Asteraceae, the flowering area was calculated as a circle (formula: πr^2^). In zygomorphic/stereomorphic flowers (bilateral symmetry; e.g. *Pedicularis* spp.) the flowering area was calculated as a rectangle based on flower length and width (formula: L × W) [3]. When inflorescences produced architectures that were nearly cylindrical (e.g. *Polygonum coriaceum*) or spherical (e.g. *Trifolum* spp.), we calculated their areas as 2πrd + πr^2^ and 4πr^2^, respectively [34].

### Plant specialization indices

Over the flowering season, a total of 5855 flower visitor individuals were collected from the 101 plant species in the four communities. From the collected insect specimens, a total of 2992 specimens representing 355 insect taxa were examined for pollen loads to determine if they carried the host plant pollen grains. One to five insect specimens were chosen from each plant-flower visitor pair at each survey and community for pollen analysis. Each pollen sample was viewed under a Hitachi S-4800 scanning electron microscope. Pollen grains were identified by comparing them to a reference library of pollen based on grains removed from field-collected flowers. If one of the specimens of a plant-flower visitor pair carried the host plant pollen we presumed that all the remaining specimens in that insect’s morphotype were also effective pollen vectors. If all the specimens of a plant-flower visitor pair failed to carry the host plant pollen, we presumed that this insect morphotype was ineffective as a pollen vector on that particular plant species. However, as we did not test the pollination effectiveness of a flower visitor by experiments such as analyzing pollen deposition on stigmas per visit [35], pollen vectors recorded in this study must be regarded as putative or prospective pollinators.

We constructed a weighted plant-pollen vector network for each survey in each of the four communities by excluding visitation interactions made by inefficient flower visitors. This resulted in a reduction of 6.3–30.4 % of total interactions for each survey of the four communities (Zhao et al. unpublished data). We calculated two plant specialization indices for each plant species in the 36 plant-pollen vector networks (4 communities × 9 surveys): pollen vector diversity (Shannon’s diversity index) and selectiveness (d’ index). Shannon’s pollen vector diversity for each plant species was calculated as ${\text{H }} = \sum\nolimits_{\text{k = 0}}^{\text{n}} {{\text{p}}_{\text{i}}   {\text{ln p}}_{\text{i}} }$, where p_i_ is the proportion of visits by pollen vector i to the focal plant species [36]. In this study, we used the total number of pollen vector individuals on a plant species to calculate p_i_. The values of a plant Shannon’s diversity index increased with the number of species and evenness of pollen vectors. The d’ index (selectiveness) expressed the relative deviation in the actual interaction frequencies of a focal plant species from a null model which assumed that all pollen vectors were used in proportion to their availability [37]. Its value ranged from 0 (minimum selectiveness) to 1 (maximum selectiveness). According to this specialization index, a high selective plant species is characterized by little overlap in its pollen vector exploitation with its co-occurring and co-blooming species. Both specialization indices were calculated in the bipartite package [38] in R [39].

### Statistical analysis

We tested for the effects of elevation, floral traits, and pollen vector community composition on plant specialization. Specialization indices and floral traits of plant species with related phylogenies may be similar due to common ancestry and hence are not statistically independent. To account for phylogenetic non-independence, we applied phylogenetic generalized linear mixed models (PGLMM) with Markov chain Monte Carlo techniques (MCMCglmm) [40]. This approach allows control for phylogenetic co-variation among species by implementing the phylogenetic tree as a random factor into the model [40]. A maximum likelihood phylogeny for all the plant species of the four communities was reconstructed from DNA sequences of internal transcribed spacer (ITS), ribulose-bisphosphate carboxylase (*rbc*L) and Maturase K (*mat*K) (Zhao et al. unpublished data).

We tested for differences in plant specialization indices (pollen vector diversity and selectiveness) among the four study communities, with individual plant species as the sampling unit. We treated elevation as a categorical fixed factor, and included survey, species identity and phylogeny as random factors. Then we tested the effects of floral traits (flower shape, flower size, floral display, flowering duration and flower abundance), pollen vector community composition (pollen vector richness and pollen vector density), as well as their two-way interactions on plant specialization. We used community identity, survey, species identity and phylogeny as random factors. We removed non-significant interaction terms by backward elimination. Models were compared based on deviance information criterion (DIC), with ΔDIC values >2 taken to indicate a significantly improved model fit [40].

For all PGLMMs, we used an inverse-Wishart prior (V = 1, nu = 0.002) for random effects according to the package guidelines (MCMCglmm Course Notes; https://cran.r-project.org/web/packages/MCMCglmm/vignettes/CourseNotes.pdf). The PGLMM models were run for 5,000,000 iterations with a burn-in of 10,000 iterations and a thinning interval of 500 iterations. Prior to all analyses, all continuous response and predictor variables were scaled to a mean of zero and a standard deviation of 1 to allow the use of regression estimates as effect sizes [41]. Estimates of the posterior mean with 95 % credible intervals (lower and upper CI) and *P* values (*P*_MCMC_) were reported. Associations between two variables were considered significant when the 95 % CI excluded zero, and *P*_MCMC_ ≤ 0.05.

## Results

There were 101, insect-pollinated, herbaceous species belonging to 63 genera, representing 26 families in the four communities. Plant assemblages included 40, 30, 33 and 27 plant species from YSZ to DYZ, respectively.

Of the 355 insect taxa collected from the four communities, 328 carried the host plant pollen and were assumed to be the effective pollen vectors. These pollen vector taxa belonged to 51 families in five insect Orders. The number of pollen vector taxa in the study communities was 163, 121, 85 and 56 from YSZ to DYF, respectively. Ten functional groups (according to presumed similarities in the selection pressures on floral traits pollen vectors exert) were detected for each community. Pollen vectors were mainly comprised of long-tongued bees and beetles at YSZ. Pollen vectors at HLG were dominated by hover flies (Syrphidae) and long-tongued bees. At YK and DYF the majority of pollen vectors were short-tongued, muscid flies (Muscidae) and long-tongued bees (Table 1).

**Table 1:** Pollen vector assemblages at four communities on the Yulong Mountain, SW China

Functional groups	YSZ	HLG	YK	DYF
Long-tongued bees	20.1	19.8	27.1	31.2
Short-tongued bees	9.8	9.5	13.2	7.8
Other hymenoptera	5.2	7.6	3.2	6.9
Muscoid flies	12.1	16.1	30.8	37.4
Hover flies	14.2	34.1	12.4	5.7
Beeflies	4.8	1.1	0.1	0.0
Butterflies	13.8	3.0	2.6	0.5
Moths	0.5	0.2	1.2	3.6
Beetles	19.2	8.0	0.4	1.4
Hemiptera	0.4	0.5	8.9	5.6

Numbers represent the percentage of visits conducted by each pollen vector functional group in each community

*YSZ* 2725 m above sea level, *HLG* 3235 m above sea level, *YK* 3670 m above sea level, *DYF* 3910 m above sea level

### Variation of plant specialization indices among communities

**Fig. 1:**
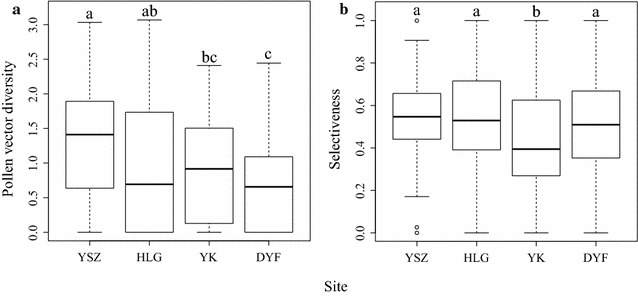
*Boxplots* of plant specialization indices at different communities on the Yulong Mountain, SW China. *YSZ* 2725 m above sea level; *HLG* 3235 m above sea level; *YK* 3670 m above sea level; *DYF* 3910 m above sea level. The *bottom* and *top* limits of each *box* are the lower and upper quartiles, respectively. The *horizontal black lines* across *boxes* are medians. *Error bars* represent the 95 % confidence interval of the median. **a** Pollen vector diversity (Shannon’s diversity index); **b** selectiveness (d’ index)

We found significant differences in plant specialization indices (pollen vector diversity and selectiveness) among the four communities. Specifically, the pollen vector diversity tended to be higher at the low elevation communities (Fig. 1a). Plant species were less selective in their exploitation of pollen vectors in the YK community which also had the highest proportion of unspecialized flowers (Fig. 1b).

### Effects of floral traits and community composition on pollen vector diversity

PGLMM analysis showed that plant species with unspecialized flowers had a greater pollen vector diversity than those with specialized flowers across the four communities (Table 2). Our analysis further revealed a highly significant and positive correlation between pollen vector diversity and flower size (Table 2). However, pollen vector diversity was not significantly influenced by floral display, flowering duration, flower abundance, or pollen vector richness and pollen vector density in the communities (Table 2). None of the two-way interactions between pollen vector composition and floral traits were significant indicating a similar pollen vector diversity response to floral traits among communities showing a different pollen vector richness and density (results not shown).

**Table 2:** Results of the phylogenetic generalized linear mixed model (PGLMM) for evaluating pollen vector diversity or selectiveness (d’) of 101 plant species in relation to floral traits and pollen vector community composition variables at four communities on the Yulong Mountain, SW China

Specialization index	Variable	Posterior mean	Lower CI	Upper CI	*P*_MCMC_
Pollen vector diversity	Intercept	0.165	−0.351	0.733	0.515
	Flower shape	−0.728	−1.082	−0.362	<0.001
	Flower size	0.146	0.012	0.280	0.034
	Floral display	−0.003	−0.113	0.114	0.962
	Flowering duration	0.014	−0.149	0.176	0.868
	Flower abundance	0.003	−0.201	0.197	0.978
	Pollen vector richness	0.205	−0.011	0.420	0.088
	Pollen vector density	0.062	−0.177	0.312	0.614
Selectiveness	Intercept	−0.031	−0.584	0.561	0.917
	Flower shape	−0.464	−0.841	−0.109	0.009
	Flower size	0.208	0.079	0.337	0.003
	Floral display	0.122	0.008	0.233	0.032
	Flowering duration	−0.031	−0.194	0.125	0.705
	Flower abundance	0.222	0.025	0.407	0.027
	Pollen vector richness	0.195	−0.018	0.417	0.084
	Pollen vector density	−0.416	−0.641	−0.181	0.001

### Effects of floral traits and community composition on selectiveness

The selectiveness of plant species in their use of pollen vectors was also related to flower shape and flower size. Plant species with unspecialized and larger flowers/inflorescences showed a higher selectiveness (Table 2). We found that plant species with larger floral displays and a greater flower abundance showed a higher degree of selectiveness (Table 2). Moreover, there was a significant negative relationship between plant selectiveness and pollen vector density (Table 2). In this case, plant species in communities with a higher pollen vector density showed lower selectiveness. By contrast, flowering duration and pollen vector richness had no significant effects on plant selectiveness (Table 2). In addition, all two-way interactions between pollen vector composition in the communities and floral traits were not significant (results not shown).

## Discussion

In this study we showed that there were significant differences in plant specialization indices among the four communities. Our results also indicated that the differences among plant species in pollen vector diversity were exclusively explained by flower shape and flower size. However, the differences in plant selectiveness were related not only to flower shape and flower size, but also to floral display, flower abundance and pollen vector density in the communities.

### Variation of plant specialization indices among communities

Olesen and Jordano [42] showed that the mean number of interacting pollinators per plant species decreased with increasing elevation. In this study we reported a similar finding. Plant species at lower elevations were visited by a greater diversity of pollen vectors than plant species growing at higher elevations. The decrease in pollen vector diversity for plant species with increasing elevation may be the result of a decrease in the number and abundance of insect taxa, in general, as elevation increased [43, this study]. However, it is difficult to tell whether elevation affects pollen vector diversity as our sampling was restricted to only one site at each elevation. Additional studies with a greater number of replicate sites at each elevation will be needed to confirm this current pattern of pollen vector diversity vs. elevation. This could also be tested by selecting a greater number of transects through a continuous elevation gradient.

The d’ index is supposed by Blüthgen et al. [37] to be more appropriate to compare specialization or selectiveness of species within or across networks because it has the advantage of not being affected by network size and sampling intensity. In our study, plant selectiveness showed no systematic variation with elevation. The plant species showed lower selectiveness within a mid-elevation community (YK), indicating that the overlap in pollen vector use among plant species was higher at this community. One possible reason for this pattern is that visitation by pollen vectors to plant species were high due to the high proportion of unspecialized flowers in this community (YSZ: 45.5 %, HLG: 41.1 %, YK: 78.9 %, DYF 69.2 %).

### Effects of floral traits on pollen vector diversity

Flower shape and flower size influenced pollen vector diversity in our study. Compared to species with specialized flowers, species with unspecialized flowers were visited by a greater diversity of pollen vectors. This relationship between floral shape and pollen vector diversity has also been found in Norway [3, 5, 11]. This is to be anticipated as unspecialized flowers are accessible to the vast majority of flower visitors regardless of their physical size, foraging behavior or proboscis length. In contrast, specialized flowers are far more likely to restrict access to their edible rewards (see above) and are pollinated primarily by pollinator guilds with canalized morphologies and behaviors [15, 17, 19, 44].

For flower size, a series of studies in Norway showed that flower size correlated positively with an increase in pollinator diversity [3, 11]. In our study, we also found a positive relationship between flower size and pollen vector diversity. This effect might be attributed primarily to the greater attractiveness of larger flowers and/or floral displays [3]. In addition to these floral traits, we cannot rule out that other floral cues (e.g. scent and pigmentation patterns) [5–8], not included in this study, also affected pollen vector diversity in some of our plant species.

### Effects of floral traits on selectiveness

Plant species could benefit from enhanced attractiveness to increase visitation rates by a greater diversity of pollinators. However, enhanced attractiveness could also have negative consequences if foraging bouts include visits to multiple plant species and these foragers transfer the pollen of one species to the stigmas of others [45]. Koski et al. [10] found that the positive relationship between flower size and plant selectiveness suggested that plants with larger flower sizes showed less overlap in flower visitor use compared with other species with smaller flowers. In addition to flower size, we also found that plant selectiveness related positively with floral display and flower abundance. This indicated that these floral traits could ultimately decrease overlap in pollen vector exploitation among co-flowering plant species.

Our finding that species with unspecialized flowers were more selective than species with specialized flowers contradicted the common assumption that specialized flower shapes, especially zygomorphic flowers, must always receive fewer pollen vector species [15]. One possible explanation is that some species with bilateral or asymmetric flowers (e.g. *Pedicularis, Lotus, Prunella, Clinopodium, Roscoea*) are pollinated almost exclusively by a few native bumblebees (*Bombus*) species. Such sharing of the same pollen vector by several plant species should ultimately decrease the selectiveness of specialized flowers. In these systems, however, interspecific pollen transfer is reduced by depositing the pollen of each co-blooming species on very isolated parts (e.g. head vs. dorsum of thorax vs. dorsum of abdomen etc.) on the same insect’s body [46].

### Effects of community composition on plant specialization

Plant selectiveness, but not pollen vector diversity, was related to pollen vector community composition in this study. Plant selectiveness decreased consistently with the increase in pollen vector density. Plant species in communities with low pollen vector density should be more selective than plant species in communities with high pollen vector density because interspecific competition among plant species for limited pollinator resources should reduce pollen vector overlap [47].

## Conclusions

Our combined analyses of the effects of floral traits and community composition in a species-rich region showed that floral traits play important roles in pollen vector diversity and selectiveness. Specifically, flower shape and flower size increased both pollen vector diversity and selectiveness. These findings indicate that some floral traits, that make the plants more attractive to insects, also increase the diversity of pollen vectors while decreasing overlap in pollen vector exploitation when plant species have overlapping flowering periods. Additionally, plant selectiveness was also associated with local pollen vector composition. This study can help us further to understand the effect of floral traits and community composition on specialization of plant species.

## Additional file

10.1186/s12898-016-0080-1 Raw data on plant specialization indices (pollen vector diversity and selectiveness), floral traits (flower shape, flower size, floral display, flowering duration and flower abundance) and pollen vector community composition (pollen vector richness and pollen vector density) at four communities on the Yulong Mountain, SW China.

## Abbreviations

CI: credible interval
DIC: deviance information criterion
ITS: internal transcribed spacer
*mat*K: maturase K gene
MCMCglmm: phylogenetic generalized linear mixed models with Markov chain Monte Carlo techniques
PGLMM: phylogenetic generalized linear mixed models
*rbc*L: ribulose-bisphosphate carboxylase gene
